# “Completely out-at-sea” with “two-gender medicine”: A qualitative analysis of physician-side barriers to providing healthcare for transgender patients

**DOI:** 10.1186/1472-6963-12-110

**Published:** 2012-05-04

**Authors:** John W Snelgrove, Amanda M Jasudavisius, Bradley W Rowe, Evan M Head, Greta R Bauer

**Affiliations:** 1 Schulich School of Medicine & Dentistry, The University of Western Ontario, London, ON, N6A 5C1, Canada

## Abstract

**Background:**

Members of the transgender community have identified healthcare access barriers, yet a corresponding inquiry into healthcare provider perspectives has lagged. Our aim was to examine physician perceptions of barriers to healthcare provision for transgender patients.

**Methods:**

This was a qualitative study with physician participants from Ontario, Canada. Semi-structured interviews were used to capture a progression of ideas related to barriers faced by physicians when caring for trans patients. Qualitative data were then transcribed verbatim and analysed with an emergent grounded theory approach.

**Results:**

A total of thirteen (13) physician participants were interviewed. Analysis revealed healthcare barriers that grouped into five themes: Accessing resources, medical knowledge deficits, ethics of transition-related medical care, diagnosing vs. pathologising trans patients, and health system determinants. A centralising theme of “not knowing where to go or who to talk to” was also identified.

**Conclusions:**

The findings of this study show that physicians perceive barriers to the care of trans patients, and that these barriers are multifactorial. Access barriers impede physicians when referring patients to specialists or searching for reliable treatment information. Clinical management of trans patients is complicated by a lack of knowledge, and by ethical considerations regarding treatments—which can be unfamiliar or challenging to physicians. The disciplinary division of responsibilities within medicine further complicates care; few practitioners identify trans healthcare as an interest area, and there is a tendency to overemphasise trans status in mental health evaluations. Failure to recognise and accommodate trans patients within sex-segregated healthcare systems leads to deficient health policy. The findings of this study suggest potential solutions to trans healthcare barriers at the informational level—with increased awareness of clinical guidelines and by including trans health issues in medical education—and at the institutional level, with support for both trans-focused and trans-friendly primary care models.

## Background

Transgender and transsexual (trans) people continue to experience discrimination, even in societies with advanced human rights protections. The social marginalisation of trans people is the focus of a growing body of scholarly work that draws attention to discrimination in employment, education, income, and housing [1-3]. Research shows that transphobia—the experience of discrimination based on having a gender identity or expression that varies from the sex assigned at birth—also constitutes an access barrier to medical care, both in general primary care [4-6], and in specialised areas such as mental health [7], substance abuse treatment [8], and HIV [9,10]. Barriers to access are multifactorial, and although contributing issues include frank discrimination such as verbal abuse, other more subtle processes create environments that are ill adapted to the needs of trans patients. Lack of knowledgeable healthcare providers and medical information regarding trans healthcare are cited as restrictions for trans patients seeking medical attention [1,9,11,12] These deficits are mirrored at the institutional level, where policies to accommodate trans patients are absent, or the very need for these policies goes unrecognised. Examples include the inability to schedule a hysterectomy for a trans male, or the refusal to accommodate in-patient placement consistent with a patient’s expressed gender at an institution with separate male and female wards. The concept of “erasure”—the systematic deficiency in acknowledging and validating trans identities, bodies, and experiences—provides a theoretical base to consider the informational and institutional processes through which healthcare barriers arise [13,14]. Erasure in healthcare settings may be propagated by the lack of organised services addressing trans patients’ needs. Even medical services directed at broader lesbian, gay, bisexual, and transgender (LGBT) populations may fail to properly accommodate trans patients [1,5,9,13]. This contributes to an emerging debate regarding the optimal organisation of trans medical care, particularly among primary care services. Some previous work on trans healthcare needs has favoured a “trans-focused” care model, with physicians and healthcare professionals specifically trained in services directed at a trans patient population [5,6,9,11]. Others have suggested that trans healthcare might well fit into existing generalised primary care models adapted to be “trans-friendly” [6,10,12,15].

In Canada, healthcare delivery is covered by 13 provincial or territorial health insurance programs [16]. These insurance programs are publicly funded, with each health insurance program federally mandated to provide essential medical services. Additional covered medical services vary between provinces, with private insurance available for “non-essential” services. Trans healthcare services are not formally organised in the province of Ontario, except with respect to sex reassignment surgeries (SRS). SRS were re-listed as publicly funded procedures in 2008 following a ten-year delisting period, with a planned increase in the number of provincial assessment centres authorised to provide referrals [17]. These policy-based initiatives signify that the Ontario government recognises sex transitioning treatments as medically necessary services, although access within the province may remain a challenge, especially outside of large urban centres. Additionally, access is not uniform across Canada. For example, the province of Alberta delisted SRS as covered procedures in 2009, and will only finance surgery for patients who had already commenced the assessment process [18,19]. Other aspects of trans healthcare, including medical transitioning treatments and non-transition related care, are not uniformly organised in Ontario.

Systematic deficiencies in addressing trans peoples’ needs may contribute to social erasure of trans bodies, with the structuring of healthcare delivery playing a significant role in how governments recognise and accommodate trans patients. Additionally, the way in which medical and mental health professionals identify and acknowledge transgenderism might further propagate healthcare access barriers for trans people. These barriers arise when eligibility criteria for specific treatments are based on a specific diagnosis, and also when patients perceive discordance between their lived experiences and a nosological interpretation of these experiences as a medical or mental disorder. In Canada, the tool used predominantly by psychiatrists and other physicians to guide diagnosis of mental health conditions is the Diagnostic and Statistical Manual of Mental Disorders (DSM), currently available as a text-revised fourth edition [20]. Transgender patients generally receive the diagnosis of gender identity disorder (GID), which is defined as a strong, consistent cross-gender identification accompanied by persistent discomfort with the assigned sex that is not based solely on a perceived cultural advantage of adopting the opposite sex, and not better explained by other psychiatric diagnoses [20]. Some researchers and members of the trans community have challenged the nosological premise that transgender identity represents psychiatric pathology. Lev (2005) describes how the DSM does not allow for a healthy, functioning variant of transgenderism, and only categorises transgender experience in the context of dysfunction [21]. Additional concerns relate to the practice of establishing GID as a precondition for medical treatment [4]. Dominant narratives of transgender experience are reinforced by the DSM criteria for GID, but these exclude other gender variant individuals for whom transitioning treatment may be of benefit. An ethical implication extending from this is the possibility that patients feel compelled to provide disingenuous histories of their transgender identity in order to ensure treatment, should their actual experiences not align with GID criteria [21,22]. The GID diagnosis often forms a part of medical and surgical transition related care, and may also influence how physicians and healthcare professionals provide non-transition care to trans patients. A formal diagnosis of GID is not considered an absolute requirement to initiate sex transitioning therapy in clinical guidelines [23-25]. Nonetheless, sex transitioning therapies are generally preceded by a GID diagnosis [24], and clinical guidelines do advise assessment of readiness to transition by a clinician with competency in mental health [23-25]. For this reason, the interaction between psychiatrists and the mental health system and physicians in other medical fields becomes important when considering healthcare barriers for trans patients.

Despite a broadening literature on healthcare barriers as identified by members of the trans community, a corresponding inquiry into healthcare provider perspectives has not been assembled. Provider perspectives do not necessarily align with those of service users, and expectations regarding the accessibility of healthcare and the outcomes of medical treatment may be dissimilar between these two groups [26,27]. In particular, there is a paucity of information about barriers to the provision of medical care for trans patients. Preliminary work suggests that lack of training, limited medical knowledge, and scant access to information sources are likely contributors to physician-side healthcare provision barriers [13,28]. Some work has been done to address medical knowledge gaps in the management of care for trans patients. Canadian guidelines have been published by Toronto’s Sherbourne Health Centre and Vancouver Coastal Health, both of which cover the dosing, contraindications, and monitoring of hormone therapy in primary care settings [23,24]. Internationally, the World Professional Association for Transgender Health (WPATH, formerly the Harry Benjamin International Gender Dysphoria Association) articulates standards of care which are considered the touchstone professional consensus around management of GID [25]. The WPATH standards set out guidelines for diagnostic assessment, psychotherapy, real-life experience, hormone therapy, and surgical therapy [25]. Real-life experience in the subjective gender is considered with reference to the social roles occupied by the individual in their vocational/educational, recreational, and family life, and the legal status of their name and gender. Parameters are given for evaluating how well a patient will adapt to their subjective gender [25].

Previous work has suggested that physician-side barriers to healthcare for trans patients include issues surrounding medical knowledge, the role of mental health services and GID diagnosis, and the organisation of health systems. The aim of this project was to expand the current literature by examining these and other barriers to healthcare provision faced by physicians when caring for trans patients. Our objective was to undertake an exploratory analysis of what these physician-side barriers are, and how they may begin to be addressed.

## Methods

This study was conducted using a qualitative approach based on grounded theory to explore physician perceptions and experiences. Grounded theory allows for the inductive generation of theoretical frameworks through systematic collection and analysis of qualitative data [29,30]. This methodology was considered appropriate because little is known empirically or from theory about physician perceptions of barriers to care for trans patients.

Participant recruitment for the study initially used purposive sampling to identify physicians in Southwestern or Southern Ontario, Canada. Given the exploratory nature of this study, practitioners from both general and speciality backgrounds were included rather than focussing on identifying issues specific to any one discipline. Physicians with little or no known contact with trans patients were not excluded from the study. Trans patients come into contact with physicians from various clinical backgrounds, and physicians without prior trans exposure may perceive care provision barriers that differ in important ways from those with trans patient exposure. Physicians with a potential interest in trans health issues or an openness to accommodating trans patients in their practice were also identified by non-medical contacts at a Southwestern Ontario trans support group. Additional participants were solicited during interviews using a snowball sampling approach, as physicians who deliver healthcare services to trans patients might be considered knowledgeable about colleagues with similar practice interests. After an initial iteration of data collection and analysis, we sought further participation using Glaser and Strauss’s concept of theoretical sampling [29]. The purpose of theoretical sampling is to provide deeper understanding of a concept under investigation. This allows for elaboration on concepts and themes in the development of a robust analytical frame—a process which ideally results in theoretical saturation of the overall conceptual framework [29,31]. In practical terms, this involved the recruitment of physicians in obstetrics and gynaecology, psychiatry, and endocrinology to elaborate on emerging themes that were underdeveloped, as trans patients are often referred to these specialists during medical transitioning. Recruitment was stopped once data phrases no longer contributed novel themes or ideas to the analytical categories.

Individual interviews were conducted by telephone or in person by one of four medical student researchers. We used a semi-structured interview template (Additional file 1) and interviewers prompted for elaboration during interview sessions, but focused on physicians’ responses in the context of their own interpretation of the issue. Semi-structured interviews were chosen to allow for a natural progression of ideas and because the chief interest of the study was perceptions and experiences of individual practising physicians. Interviews were audio-recorded and transcribed verbatim, with personal identifiers removed. A participant’s gender and medical specialty were reported and linked with quotation excerpts following their consent. Informed consent was obtained in writing for in-person interviews and verbally recorded for telephone interviews. Participants were informed that they could withdraw consent to participate at any time during the interview or thereafter should they choose.

Transcript files were imported to an open-source qualitative analysis program, Weft QDA 1.01 (http://www.pressure.to/qda/) for analysis. Data phrases from interviews encompassing a complete concept or idea were tagged. We then coded these data phrases using an emergent approach [31]. Thematic areas were identified on a continual basis, whereby categories were compared and grouped with one another. Data phrases that articulated contrasting opinions about the same topic became incorporated into the same category to capture a breadth of perspective. Results were circulated back to participants for verification. The University of Western Ontario Ethics Review Board provided ethical approval for this project. The study design and analysis adhere to the RATS guidelines for qualitative research [32].

## Results

### Participants

Thirteen (13) physicians participated in this study. The majority (9) were general practitioners, with practice characteristics ranging from comprehensive family medicine to population-specific LGBT health and HIV care. The remaining participants practised in the specialties of obstetrics and gynaecology, endocrinology, nuclear medicine, and psychiatry. The geographical distribution of participants was focused in urban and small-city settings, although two had rural practices. Approximately half were affiliated with an academic centre. Participants ranged in age from 29 to 57. Seven (7) identified as male and six (6) as female; none identified as transgender. Five (5) participants had no known experience directly caring for a trans patient, and five (5) had up to five trans patients in their practice at the time of interview. Three (3) participants—one psychiatrist and two family physicians—described trans patients as a significant proportion of their practice population. Participants who knowingly had contact with a trans patient in their practice were able to offer more complex accounts of care barriers than those without similar exposures. This reinforced the observation that contact with a trans patient provides an instigating event for physician reflection and education on the healthcare needs of this patient group.

### A centralising theory: “Didn't know where to go or who to talk to”

Physicians commonly identified barriers to care provision in the context of not knowing the available resources or care strategies appropriate for the trans patient population. This overarching concept persisted across other major themes and provided a focus for the analysis. Participants with no experience caring for trans patients as well as those with experience expressed similar concerns, as demonstrated respectively in the following excerpts.

""I have wondered if a patient like that [trans] was to come along what I would do. And the answer is I have no idea… I mean if a patient was looking for sex reassignment surgery or something along those lines, I would have no idea who to send them to." Dr. T, family physician, male."

"“…despite trying to find ways to improve my expertise, I just didn’t know where to go or who to talk to, or where to get the information, and I felt really bad because some of my initial attempts to help these people—I sent them to people I wish I hadn’t sent them to.” Dr. S, family/HIV primary care physician, male."

Categories that emerged during the analysis elaborated on the barriers physicians face when providing care to trans patients. These categories clustered into five (5) related but distinct thematic areas: accessing resources, medical knowledge deficits, ethics of transition-related medical care, diagnosing vs. pathologising trans identity, and health system determinants.

### Accessing resources

A major barrier to healthcare provision was the identification, availability, and quality of referral networks and information sources regarding trans medical care. Identifying “trans-friendly” colleagues for referral outside of one’s scope of practice was difficult, both for lack of knowledge regarding specialist availability, and for concern regarding colleagues’ sensitivity. SRS referrals were chiefly concerning given the highly specialised skill set required for these therapies. Depending on the procedure, surgeons capable of performing SRS may not be available regionally, forcing referring physicians to consider alternative options.

"“I have felt completely out-at-sea without a life preserver in terms of trying to find physicians, both nationally and internationally, who can assist in terms of their surgical transition.” Dr. S, family/HIV primary care physician, male."

Reasons for insensitivity toward trans people were understood mainly in moral terms—recognising that some physicians could have entrenched personal beliefs about gender identity, sexuality, and sexual health that are at odds with trans patients’ lives. Participants varied in how they characterised these biases, but most described physicians’ comfort with trans people in dichotomised terms.

"“Doctors can kind of go two ways right? You can either see a whole bunch of life and decide that there’s no point in being judgmental—everyone’s different and, you know, everyone’s got their own path in life and not to judge people—or you can just get really narrow-minded and somehow build this construct between ‘us’ and ‘them’, with ‘them’ being the patients.” Dr. W, family/HIV primary care physician, male."

This reflected a general perception that certain clinicians will be interested in trans healthcare and include this in their practice, and other physicians will not. Physicians employed two main strategies to deal with referral issues concerning colleagues’ sensitivity. In the “hit or miss” approach, referrals were made based on patient care needs without knowing how sensitive the consulted physician would be. Alternatively, referrals were directed to physicians with known interests in caring for sexual minorities, such as physicians with large homosexual practice populations. Patient feedback was an important determinant of future referrals.

"“But now, really throughout all the hospitals you know, there’s good people there and it’s trial and error, like you know you refer somebody and they come back and say, ‘you know that guy was a total dickhead’ or ‘you know he treated me like I was from another planet’ and so you just know not to refer to those people again.” Dr. W, family/HIV primary care physician, male."

The complexity of a physician’s referral strategy appeared to be related to their level of experience in managing the care of trans patients. Those with more familiarity in trans healthcare referred patients to specific colleagues with whom they had developed a working relationship.

Overwhelmingly, participants cited deficiencies in informative sources regarding trans care management as a substantial barrier to care. Most participants specified the need for readily accessible information through efficient media, such as the internet. The intended audience for information sources was also considered, since physicians may not consume information unless it is directed toward them explicitly.

""It’s going to, however, have to be targeted to a physician, because we need that. We need to feel that we’re getting different information than the public." Dr. A, obstetrician/gynaecologist, female."

Others suggested that the validity of information might be judged by the media used to distribute it. Academic journals, internet sites endorsed by a professional medical organisation, and Continuing Medical Education (CME) conferences were considered more appropriate sources than general information found on the internet. One participant noted that CME activities may be credited toward ongoing license maintenance requirements. While still seen as deficient, according to some participants the availability of trans healthcare information has increased since entering clinical practice.

### Trans-specific medical knowledge deficits

Barriers concerning physicians’ clinical experiences with trans patients mainly related to knowledge deficits. The lack of advance exposure to trans patients prevented physicians from acquiring clinical proficiency in trans healthcare needs.

"“I would imagine that the average physician, especially if you’re a family doctor, has had no exposure to these sorts of things. You know, the first time you get a transgendered patient come into your office, you’re sort of lost and you don’t really know what to do.” Dr. K, nuclear medicine physician, male."

Additionally, formal education around trans healthcare was described as absent from medical school and residency curricula. Despite agreeing on the lack of trans-related education, participants differed in opinion on strategies to ameliorate training deficiencies. For some, medical school education should incorporate issues pertaining to trans healthcare.

"“I think one issue that concerns me is the lack of training and/or exposure that med students and residents have to the transgendered population and indeed the general population of people, you know often will think that they’ve never met a trans person. Well many of them have. So you know the feeling that, ‘well this doesn’t affect my practice or my life,’ you know perpetuates the lack of, the idea that there’s just no need for training around trans issues.” Dr. M, family physician, female."

In contrast, the idea that medical school curricula should focus on more commonly encountered conditions was also presented, suggesting that transition-specific medical care could be covered through clinical exposures and supplemental learning opportunities based on the individual interests or practice needs of physicians.

"“I think at the level of medical school when you’re trying to learn like this much [wide arm gesture], that, you know, I’m not sure that it’s something there is room for…"

"… I don’t know whether that’s the best use of time. You always are going to learn more about what you need to learn or want to learn. Just do the research.” Dr. Y, family physician, female."

Four different strategies to ameliorate training deficiencies emerged from the analysis. Three of these were discussed in favourable terms: physician support networks, explorative interest, and clinical guidelines. One additional strategy was noted as an unfortunate consequence of few physicians being comfortable managing trans healthcare, and detailed how trans patients are often left to research referral and treatment options for themselves.

"“What I’ve had to do is rely on the patient to tell me how best to manage, and not that I’m adverse to that, I’m really happy, but it was really the community themselves who have nested and have isolated, you know, where should somebody be sent under these circumstances, where should they be sent for this issue. And so I really appreciated that the community themselves have mustered up that internal support and guide physicians, but that’s pretty suboptimal when patients have to tell doctors, you know, how to do what we should know how to do.” Dr. S, family/HIV primary care physician, male."

Physicians compared the consultation support provided for cardiac conditions and HIV care with what could be established for trans healthcare needs. They suggested that experts in the area could support colleagues with less experience through physician networks. Another strategy identified the potential for physicians to become more informed through an explorative interest in the area. The field of trans healthcare was seen to provide unique practice opportunities for physicians, particularly in psychiatry and endocrinology. Although a physician may not commonly encounter trans healthcare issues, these are not necessarily outside their scope of practice. An example of this was given: endocrinology training in hormone replacement does not usually include hormone therapy for trans patients, but the knowledge base of an endocrinologist is sufficient to accommodate trans patients requiring hormones for transitioning. Lastly, clinical guidelines emerged as a potential solution to knowledge deficits. Participants with marginal or no experience caring for trans patients identified the usefulness of these resources, but were unaware of guidelines that are currently available.

"“For physicians to have access to guidelines as to therapy, hormonal therapy you know replacement therapy but also, like I said, the interesting things that you have to remember; For example, the prostate may still be in place even though they’re now female, and those kinds of things you need to remember. And having some sort of guidelines for that I think would be of real benefit.” Dr. L, endocrinologist, female."

Participants with significant trans patient populations mentioned existing guidelines for management of medical transitioning, underscoring the importance of flexibility in their application.

"“The Harry Benjamin guidelines [WPATH guidelines] are still like the key guidelines and, like I say they’re not a bad starting point but you do have to add a bit of common sense to it.” Dr. W, family/HIV primary care physician, male"

Guidelines themselves were seen to present potential barriers when their criteria limited treatment options or prolonged a patient’s treatment course unnecessarily. The issue of “wait time” in medical transitioning was important for participants experienced with hormonal therapy and surgical referrals. They noted that guidelines can include a chronological treatment framework. While this ensures patient readiness for medical transitioning, the length of wait time needed was seen to vary based on individual patient factors. One participant noted that some physicians follow chronological criteria exactly, while others take a more flexible approach and initiate hormone treatment earlier based on their clinical judgment.

### Ethics of transition-related medical care

Participants with experience treating trans patients identified several barriers related to the unique clinical relationship between trans healthcare needs and the medical system—the nature of which is distinct from those of sexual minorities. Trans people often rely on hormones and surgical procedures to reconcile their anatomy and physiology with their gender identity, which relies to some extent on medical access.

"“Homosexuality doesn’t need medical treatment, unless trauma because of experienced or internalised homophobia, which of course needs treatment."

"…But with gender identity, because it requires a physical changing, it requires medical knowledge.” Dr. M, family physician, female."

This unique relationship presents several key issues. Given the importance of decisions surrounding medical transitioning, physicians may feel uncomfortable influencing a person’s choice to pursue treatment or not. This can have little to do with a physician’s comfort with the actual therapy and its monitoring, as illustrated by one participant who regularly prescribes hormone treatments for transitioning patients:

"“I encourage everyone to explore their gender identity, even people who are not transgender. However, I won’t encourage anyone to transition physically, or to have surgery, or to take hormones, or even to explore too much more in depth their gender identity than makes them comfortable.” Dr. M, family physician, female."

The reluctance to influence a patient’s decision to undergo treatment was almost exclusively related to concern that the patient may later regret their choice.

"“I don’t like to deny treatment and sometimes if somebody comes in and they give a very compelling history and it seems pretty obvious that they’re transgender, then I don’t want to put obstacles up. But on the other hand, you know one of my worst fears is that somebody will regret their decision that I’ve taken part in.” Dr. W, family/HIV primary care physician, male."

Participants also considered how the effects of hormone treatment differ between genetic males and females; the physiological consequences of masculinising hormones in genetic females are less reversible than feminising hormones in genetic males. Treatment regret also emerged in a medico-legal context. Physicians were cognisant of the legal ramifications of initiating therapy, potential side effects, and the communication of patient information between providers.

Patient expectations comprised a final barrier to transition-related care. Physicians noted that patients might have ideas about how they will feel and look after transitioning that are unrealistic for various reasons.

""It’s like every teenage girl coming to me and saying 'Well, I want to look like Angelina Jolie' or every guy saying they want to look like Brad Pitt… And those are terribly unrealistic expectations people have of the medical system… You know it’s a struggle for me to tell people, 'No, you know, you shouldn’t be expecting this.' And there are some people who are really out of touch with reality around it because they have seen other people transform themselves in such a drastic and a dramatic way, however that may not be possible for everyone for various reasons.” Dr. M, family physician, female."

Physicians may find it difficult to predict realistic treatment outcomes, or to ensure a patient’s expectations can be accommodated with the treatment being considered. Unrealistic expectations among physician colleagues added another obstacle to care. Even physicians with experience in providing gender transitioning therapies may not appreciate the variability of treatment outcomes.

### Diagnosing vs. pathologising

Several participants expanded on the concept of pathologising trans status—most notably through the inclusion of GID in the DSM [20]. Four main issues were identified. First, healthcare professionals often confuse GID with other psychiatric diagnoses—the paraphilia known as transvestic fetishism, for example. The fact that the paraphilias also cover mental illnesses predisposing an affected individual to criminal activity—such as the case with paedophilia—was of particular concern. A second issue was that providers often assume trans patients have other psychiatric conditions, or that having GID necessarily puts a patient at higher risk of other mental illness.

""Most of them [trans patients] don't have any other diagnoses and I think there's a misperception among a lot of medical people—psychiatrists and others—that it's so not within the normal range of what they understand that they just assume that these folks must have other significant pathologies, which is just not true." Dr. N, psychiatrist, female."

A third issue related to accessing treatment for patients who medically transition. Access to healthcare is historically premised upon identifying a condition that requires treatment. This has implications for the public funding of recognised medical treatments and in identifying the medical practitioners who should be responsible for the care of certain populations. A potential benefit of including trans identity in the DSM is that its codification makes it a medically recognised condition requiring attention from providers.

"“I guess if you're removing something from the DSM, well where are you going to put it so that people can still get the services that they need—in this case, hormone treatment and sex reassignment surgery? So who's going to look after that, if not psychiatrists right? …So before removing it [from the DSM], which I think should be done at some point, I agree it pathologises what should not be pathologised, but it's still something that requires treatment, right? So who's going to do the treatment? Which means the decision maker is not going to be a psychiatrist. That would have to be worked out before it would get removed.” Dr. N, psychiatrist, female."

A final consideration was how trans status—and GID in particular—should be dealt with as a psychiatric diagnosis. While participants generally agreed that a trans identity is not itself pathological, its inclusion in the DSM—and, broadly, its categorisation as a psychiatric condition—evoked contrasting ideas.

""The best thing would be to … make it its own category, and I would maybe call it ‘Core conditions related to gender identity,’ which are generally not, first of all, pathological." Dr. M, family physician, female."

Some participants advocated for reclassification of GID within the DSM, while an alternative opinion was that GID should be removed from the DSM altogether.

### Health system determinants

The concept of “two-gender medicine” emerged to characterise health system barriers. At the institutional level, these barriers manifest as systematic failures in recognising and accommodating the healthcare needs of trans patients.

"“…So I looked at this gentleman and thought, what room am I going to put you in? Like, because if you’re in a room with men—which you deserve to be—the nurse is going to come in and ask you if you’re doing fine since your hysterectomy. That’s not fair to him with the other men in the room. So I said ‘You’ll have a private room, regardless of what your health insurance says.’ So I can do that for him, as a physician looking after him.” Dr. A, obstetrician/gynaecologist, female."

Participants explained that sex-specific eligibility criteria for certain procedures, screening tests, or therapies could prevent adequate healthcare for trans patients. This phenomenon was distinct from issues concerning clinician experience or knowledge deficits. Rather, health system level barriers exist when it is impossible or difficult to order a test or therapy for a patient who is considered ineligible based on their gender.

""If I had a transsexual who needs to get a prostate ultrasound—so this is a born male but now is a female transsexual—unfortunately, that can be problematic at the lab doing the prostate ultrasound.” Dr. S, family/HIV primary care physician, male."

Where care is not hindered outright by eligibility deficits, limited care options remain a significant barrier. In the context of medically transitioning sex, these barriers stem from having few practitioners available to initiate and monitor therapy, and from restricted management options for transitioning. As an example of the former, one participant described difficulty finding a urologist who would perform an elective orchiectomy:

"“This [trans female patient] started to have some problems from [her] exogenous hormonal therapy and [she] very appropriately elected to have an orchiectomy, which would then circumvent the need for all these exogenous hormones. I had difficulty finding a urologist who would do an elective orchiectomy for this issue, but eventual did find one.” Dr. S, family/HIV primary care physician, male."

Regarding the latter, several participants described the standardised protocols at the Centre for Addiction and Mental Health (CAMH) in Toronto, Ontario, a facility that provides assessment and referrals related to medical and surgical transitioning. Participants noted that healthcare providers might feel limited in the options they can offer patients when a group standard is in place. More concerning was the fact that some health institutes actively discouraged providers from using clinic time for trans patients.

""I do have to do this after hours for a reason because my employer, shall we say, would not allow me to create the clinic I would like to create within the hospital system, and so doing it on my own time is the only way I can do that…"

"…So there's a lot of misunderstanding that way and a lot of, I'd have to say transphobia and definitely homophobia as well, when I suggested the idea of having an LGBT clinic within my organisation. I was told 'Well, you can do that quietly.' But you can't create a program quietly." Dr. N, psychiatrist, female."

Participants acknowledged that even where specific policies discriminating against trans patients are not in place, the general attitude of an institution contributes largely to care delivery at that site. Inadequate cultural competence and restrictive policies—whether official or not—were seen to contribute to systematic discrimination and transphobia that manifest as barriers to care provision at the institutional level.

The organisation of physician roles and responsibilities was another element influencing health system level barriers. Contrasting ideas were presented regarding the responsibility of primary care physicians in trans patient care. Some participants—particularly those in specialist areas—expressed frustration that certain health services which could be provided by primary care physicians are not.

"“There’s only one endocrinologist in the city that prescribes hormones for transgendered individuals, so the wait list to see that particular person is quite long… and we don’t have a lot of family doctors in the city that I know of that are willing to prescribe the hormones. Certainly family doctors are capable if they have the training and the knowledge, but some people are just not interested in gaining that knowledge it seems, so that's a bit frustrating. It's all gotten to the point where I've thought about getting training, but unfortunately it would probably not be a good idea for their psychiatrist to be prescribing the hormones." Dr. N, psychiatrist, female."

Service provision responsibilities were often seen to fall under specific disciplines within the medical community. However, some services—such as hormone therapy in the preceding example—were considered by several participants as appropriate for primary care physicians to provide, despite also being covered under the practice scope of specialists. A contrasting perspective was that primary care should focus on general healthcare, making use of referrals for more complicated management issues. The following example demonstrates this point and how, for some primary care physicians, hormone prescription might be viewed as a more specialised service not to be provided without specialist consultation.

"“But it also comes from, you know, what do trans patients need? You know if you’re not comfortable doing the hormones you can always refer to an endocrinologist. But apart from that what they need is they need to have their blood pressure checked, if they’re over 55 they need colonoscopies, they need physicals once a year… They’re people right, first and foremost they’re people. So you know, I’m not comfortable dealing with complicated cardiac arrhythmias or deciding to start people on amiodarone. I don’t do that, so I refer that part to a cardiologist and I take care of the rest of the person. That’s what primary care does." Dr. W, family/HIV primary care physician, male."

These divergent opinions reflected different expectations regarding which services should be provided by the primary care system. While it was generally recognised that family medicine should encompass general care issues for any patient—including trans patients—some participants suggested that primary care services targeted specifically to the trans population in a “trans-focused” care model would be more effective. These participants reiterated that it may be harder for trans patients to obtain appropriate care under a generalised but “trans-friendly” primary care system for reasons related to patient concerns, physician bias, and other barriers described previously.

The unique position of psychiatry in addressing trans healthcare needs emerged in two main areas: first, regarding consultation prior to hormonal or surgical transitioning and second, in instances where a patient with a mental illness requires professional consultation and happens to be trans. The role of the psychiatrist in the treatment consent process was framed in non-exclusive terms. Other physicians involved in the care of a trans patient may just as appropriately assess patient competency in making treatment decisions. However, in practice, physicians seem reluctant to obtain consent without consulting psychiatric services.

""And certainly, I can see why it's important to have a psychiatrist rule out some specific conditions before changes are made that are irreversible, such as hormone treatments and gender reassignment surgery. I absolutely understand why it's important to rule out some of those things, but in terms of, you know, should psychiatry be the only specialty that has the opportunity to say, 'Yes, this person should be qualified for the surgery?' Probably not. I would imagine that endocrinologists, for example, or even family doctors with special training could probably make those kinds of decisions without a psychiatrist. But we're not there yet." Dr. N, psychiatrist, female."

The notion that gender identity constitutes a special area of psychiatry was cited as a barrier, considering the few psychiatrists who work in the area.

"“So it’s even worse for transgender patients because they’ve got to deal with the general shortage of psychiatrists and a lack of funding for a broader spectrum of psychological services. In addition, even if they get in the door to see a psychiatrist, most psychiatrists are going to say, 'Oh I don’t know how to deal with this, you have to go and see the experts.'” Dr. W, family/HIV primary care physician, male."

This became particularly important outside of transitioning treatment, because gender identity was described as overemphasised in the evaluation of trans patients with a mental illness.

## Discussion

The findings of this exploratory qualitative study show that physicians perceive significant barriers when providing healthcare services to trans patients. The central theme that emerged emphasised uncertainty in multiple areas of healthcare provision. Access barriers impede physicians during patient referrals, and when searching for reliable information. The clinical management of trans patients is complicated by a lack of knowledge and experience, and by ethical considerations regarding medical transitioning treatments, which may be unfamiliar or challenging to physicians. The process of diagnosing GID might further complicate care, particularly regarding access to transition-related treatments. At the health system level, the disciplinary division of responsibilities in the medical profession poses additional care barriers, and policy to address healthcare service deficits is underdeveloped. While significant barriers exist, participants in this study, from various clinical backgrounds and practice settings, were able to describe potential solutions. These included improved sources of information (such as clinical guidelines), increased dissemination of current guidelines, incorporation of trans healthcare issues into medical curricula, better collaboration with knowledgeable colleagues, and policy-based initiatives to improve access to healthcare services for trans patients.

This analysis suggests that the traditional referral process used for specialist consultation contributes to barriers in two main ways. First, physicians may have difficulty in identifying colleagues with sufficient expertise to address the clinical problem, or they may worry about the sensitivity of colleagues when referring a trans patient for specialist care. The patient-side literature on trans healthcare barriers reflects a similar concern over limited access to knowledgeable, sensitive clinicians [1,6-8]. Second, relying too heavily on specialised services favours the notion that trans patients comprise an exceptional subgroup, rather than a primary care population with a set of care needs that are not limited solely to medical transitioning. Physician uncertainty with care options and referrals was usually attributed to information deficits. Previous research has shown that healthcare providers for LGBT patients have unique informational needs, and desire access to reliable LGBT specific resources [28]. The uncertainty of patient satisfaction was an important element linking information access and the ethics of transition-related care. Participants with experience in gender transitioning treatments expressed concern that patients may be unsatisfied with results or, worse, altogether regret their decision to undergo treatment. Perspectives from the trans community demonstrate a similar concern over lack of information on long-term outcomes and potential complications of gender transitioning treatments [13]. The literature on patient satisfaction after surgical transitioning is not extensive, but suggests that patient regret is relatively minimal, with a prevalence ranging from 2-6 % depending on the procedure [33-35]. Clinical guidelines were seen as a way to address knowledge deficits in gender transitioning treatments and preventative screening, but were not without criticism. In particular, the most recent WPATH standards at the time of interviews suggest three months of real-life experience before initiating hormone therapy [25], which is problematic for patients who feel uncomfortable living in their subjective gender without some of the physical changes induced by therapy. However, guidelines do give additional consideration to the initiation of therapy without definitive GID diagnosis under a harm reduction model for those who would otherwise use illicit hormones, and for patients experiencing significant distress regarding their gender presentation [23-25]. The availability of clinical guidelines online has increased their accessibility, but more work needs to be done promoting these resources among clinicians. The formation of physician support networks emerged as another solution, addressing issues with referral and knowledge deficits. Informal networks already exist, where primary care physicians have identified trans-friendly specialist colleagues, and where rural physicians have consulted urban colleagues with more experience managing hormone therapy. However, a cohesive professional network of physicians providing trans healthcare does not yet exist in Ontario. Such a network may reduce barriers related to the referral process by making health services for trans patients less dispersed and increasingly available at one-stop clinic locations, a concept that has been shown to appeal to the trans community [1,5].

The interaction between gender identity and psychiatric diagnosis became an important topic in the present study. A shortage of physicians (psychiatrists and other) competent in diagnosing GID contributes to treatment delays and restrictions. Overall, participants in this study recognised benefits and drawbacks to the inclusion of GID as a mental health condition in the DSM. On the one hand, the codification of transgender identity supports the autonomy of patients by validating their gender experience within the medical community, and by establishing diagnostic criteria through which a person may access treatment. On the other hand, the diagnosis of GID is restrictive in its applicability to only dominant transgender variants and pathologises transgender experience as a disorder. Patient-side research has likewise identified an overemphasis on trans status in mental health evaluations of trans patients, and concern about the “gatekeeper” role of mental health professionals for access to transitioning treatment [1,4,13]. Participants who discussed the role of diagnosis in gender transitioning articulated these competing perspectives, but were in favour of retaining some aspect of diagnosis. Previous work on trans identity erasure provides insight for why this may be. Namaste (2000) argues that the acceptance of psychiatry’s role in classifying gender—for example, with the diagnosis of GID—legitimises this discipline in both regulating and defining transsexuality [14]. While this is problematic, Namaste (2000) describes the potential for “reverse discourse,” whereby trans individuals uptake the vocabulary used to describe them in psychiatric and medical contexts to organise for political resistance, or to advocate for certain rights. An extension of this explains how acceptance of GID as a psychiatric diagnosis may provide avenues for patients to gain better access to healthcare. Dewey (2008) found that trans patients supported the established discourse regarding gender identity through the use of medical language in attempts to access transitioning treatments. Use of medical language has also allowed patients to engage in resistance activity by challenging medical decisions [22]. Previous work suggests that the process of diagnosis confers medical legitimacy and advances the treatment needs of trans patients as clinically necessary services rather than cosmetic therapies [21,36]. Physicians might feel that a DSM diagnosis offers greater likelihood of treatment accessibility than alternative options, and the literature implies that in a similar way, trans patients might support GID as a diagnosis if it eases access to treatment. Notably, an updated fifth edition of the DSM is scheduled for release in 2013, with proposed changes to the name (from GID to gender dysphoria) and diagnostic criteria for the condition [37]. These changes will likely advance a less pathologising conceptualisation of trans identity, although it is unclear whether changes to DSM criteria and diagnoses will be reflected in clinical guidelines, or if the “gatekeeper” role of psychiatry will change significantly for access to transitioning treatments. Of note, the recently revised WPATH guidelines distinguish between gender dysphoria and gender nonconformity and call for a “de-psychopathologisation” of trans identity [38]. These revised guidelines became available after the data collection for this study.

Access, sufficient medical knowledge, a thorough understanding of potential ethical considerations, and the issues associated with GID diagnosis all affect how care is provided by physicians to trans patients. These barriers also raise the issue of how service provision is best modelled. In this study, a contrast emerged between a trans-focused care model versus a trans-friendly—but otherwise general—primary care model. Most participants advocated for generalised competencies under a “trans-friendly” model for trans medical knowledge, the basics of medical transitioning assessment and treatment, and overall healthcare issues relevant to the trans population. However, this was not unanimous. This contrast is reflected in the literature, with arguments in favour of trans-focused care models [5,6,9,11], and also generalised trans-friendly models [6,10,12,15]. Part of the difference in opinion likely relates to the complexity of trans patients’ healthcare needs. This analysis found evidence that the determinants of such needs extend beyond medicalised distinctions separating “general” and “trans-specific” healthcare issues. Although not direct barriers to healthcare provision, participants recognised that social inequalities, poor social support, and discrimination unavoidably influence healthcare access for trans patients. In this way, social exclusion propagates the healthcare barriers experienced by trans patients. One emergent concept characterised the trans community as a “hidden population”—a group underestimated in size and therefore underrepresented in social spaces. Several participants correlated the lack of knowledge in trans healthcare with the relative obscurity of trans identities within the general population. While trans individuals undoubtedly represent a minority, existing population estimates are considered inaccurately low [13,39], and a recent U.S. estimate posited that 0.3 % of adults are transgender [40]. The assumption that most physicians will never encounter a trans patient contributes to informational erasure, whereby the need for healthcare training, research, and policies inclusive of trans people is systematically unrecognised [13]. In consideration of this, several participants advocated for inclusion of trans healthcare topics in medical curricula, or introducing medical students to trans people early-on during their education. These strategies, and improved familiarity with existing clinical guidelines, could address healthcare barriers by normalising trans identity amongst medical trainees. Other participants described the “hidden population” phenomenon differently, suggesting that clinical encounters with trans patients are more common than generally acknowledged, but for various reasons, trans patients may be unwilling to disclose their transition history to physicians. The literature on trans patients’ experiences with healthcare professionals offers insight into the factors that promote or discourage openness about gender identity. A holistic approach to medical care, perceived interest in patient wellbeing, and the ability for patients to identify according to subjective gender rather than as necessarily “transgender” all encourage unrestrained patient-provider communication, whereas perceptions of poor provider knowledge or insensitivity towards transgenderism are clearly obstructive to disclosure [5,9,15,22]. Participants in this study described exclusion at the institutional level mostly in terms of passive erasure, whereby policies fail to adequately accommodate trans identities [13]. Notably, one important example of active erasure was mentioned—the reluctance one participant encountered from her institution when establishing a clinic to accommodate trans patients. Trans-focused and trans-friendly care models address trans patients in relation to their healthcare needs, whether these needs are transition related or not. Presumably, either model would result in forms of reverse erasure at the institutional level by acknowledging trans patients and adapting health systems to accommodate them. The present study found support for both models but, given its exploratory nature, questions regarding which model would result in better care provision were out of scope.

Several limitations of the present study should be mentioned. Of the thirteen participants, five had no known clinical contact with a trans patient, although all were cognisant of the possibility that they had cared for a trans patient unaware. Since we were interested in perspectives on provision barriers amongst physicians with known trans patients as well as those without, no attempt was made to exclude the latter. Less experienced participants described barriers in accessing resources, medical knowledge, and those related to the organisation of the healthcare system, but often could not give specific examples. Only experienced participants provided insight into barriers regarding GID diagnosis and the ethics involved with transition-related medical treatments. Regarding participant recruitment, we initially approached physicians known by two of the authors to have an interest in trans healthcare, and physician contacts from a trans support group. Non-medical group members suggested physicians who either had expertise or interest in trans healthcare, or who were perceived to be open to trans patients. Additional contacts were solicited from the participants themselves during interviews. This presents issues around selection bias, particularly given the likelihood that contacts share similar ideas regarding healthcare barriers. The exploratory nature of the study justified the recruitment of physicians from different clinical backgrounds with varying degrees of trans patient exposure, as our aim was to identify some of the physician-side barriers to caring for trans patients and strategies for addressing these. However, because of select participant recruitment, it is likely that additional barriers and strategies remain unidentified. An additional limitation was the number of participants. Recruitment continued until thematic areas no longer expanded with additional data. However, given the small number of physicians identified as having a specific interest in trans healthcare, and the limited experiences amongst other physician participants, our study drew from a small sample size.

## Conclusions

This exploratory study provides a novel inquiry into physician-side barriers to healthcare provision for trans patients. Barriers previously identified by the trans community were found to limit care provision by physicians as well, including the inaccessibility of resources and appropriate referrals, inadequate medical knowledge and training, the limitations of GID diagnosis, and the low availability of trans healthcare services. This study presents additional insight to physician-side barriers involving the ethics of providing transition-related medical care. The findings contribute to an emerging debate regarding models of trans healthcare organisation, and how these may address some of the barriers faced by trans people and their physicians within the healthcare system. While the findings of this study elucidate some of the barriers faced by physicians, more research is needed to fully understand healthcare provision barriers, and to develop solutions that are acceptable to both the medical and trans communities.

## Abbreviations

CME = Continuing Medical Education; DSM = Diagnostic and Statistical Manual of Mental Disorders—refers to DSM-IV TR unless otherwise specified.; GID = gender identity disorder; HIV = human immunodeficiency virus; LGBT = lesbian, gay, bisexual or transgender; SRS = sex reassignment surgeries, which may include hysterectomy, oophorectomy, phalloplasty, metoidioplasty, mastectomy and chest reconstruction, orchiectomy, and vaginoplasty.

## Competing interests

The authors declare that they have no competing interests.

## Authors’ contributions

All authors contributed to the conception and design of the study, and interpretation of the data. JS, AJ, BR, and EH conducted interviews and analysed the data. JS wrote the manuscript and GB provided revisions. All authors read and approved the final manuscript.

## Pre-publication history

The pre-publication history for this paper can be accessed here:

http://www.biomedcentral.com/1472-6963/12/110/prepub

## Supplementary Material

Additional file 1 Participant interview schedule Click here for file
