# Supplementary material for: “Completely out-at-sea” with “two-gender medicine”: A qualitative analysis of physician-side barriers to providing healthcare for transgender patients
Source: BMC Health Serv Res. 2012 May 4;12:110. doi: 10.1186/1472-6963-12-110 (PMC3464167; doi:10.1186/1472-6963-12-110)
Supplement: Additional file 1 — Participant interview schedule [file 1472-6963-12-110-S1.doc]

**Semi-structured interview schedule**

**[*First obtain consent—either on paper or on record.**]**

1. **How do you describe your gender?**
2. **Into which of the following categories does your age fit:**
   1. <30
   2. 30-39
   3. 40-49
   4. 50-59
   5. 60-69
   6. 70+
3. **What is your specialty?** [open-ended, record response]
   1. [if not willing to disclose specialty] **Would you be comfortable telling me if you are a general practitioner or a specialist?** [GP/specialist]
4. **Of the following, how would you describe the location of your practice:**
   1. **Academic centre**
   2. **Urban non-academic**
   3. **Small city non-academic**
   4. **Rural community**
   5. **Other** [open-ended, record response]
5. **If we include quotes from this interview as part of our study results, may we use this information (above) to describe you?** [Yes/No]
   1. [if “no”] **If not, how should we describe you?**
6. **To your knowledge, have you ever or do you currently have transgender or transsexual patients?** [open-ended, record response and prompt for further elaboration]
7. **Are there issues with providing care to trans patients? If so, what issues do you think exist in providing general care to trans people.** [open-ended, record response and prompt for further elaboration, prompt to ensure consideration of all types of care (primary, emergency, specialist) and not just medical transition-related care]
8. **What concerns do you think physicians might have with providing care that is directly related to a person’s health care needs as transgender or transsexual?** [open-ended, record response and prompt for further elaboration—prescribing, administering, monitoring hormone therapy, referrals for transition-related services]
9. **What do you think physicians would need to support them in providing care to trans patients?** [open-ended, record response and prompt for further elaboration]
10. **Is there a special role for primary care in providing healthcare to trans people?**
11. **Is there a special role for psychiatry in providing healthcare to trans people?**
12. **Are there any other issues that you think are important with respect to healthcare for trans patients that we haven’t touched upon?** [open-ended, record response and prompt for further elaboration]
